# Collaborating on Data, Science, and Infrastructure: The 20-Year Journey of the Cancer Research Network

**DOI:** 10.5334/egems.273

**Published:** 2019-03-29

**Authors:** V. Paul Doria-Rose, Robert T. Greenlee, Diana S. M. Buist, Diana L. Miglioretti, Douglas A. Corley, Jeffrey S. Brown, Heather A. Clancy, Leah Tuzzio, Lisa M. Moy, Mark C. Hornbrook, Martin L. Brown, Debra P. Ritzwoller, Lawrence H. Kushi, Sarah M. Greene

**Affiliations:** 1 Division of Cancer Control and Population Sciences, National Cancer Institute, Bethesda, MD, US; 2 Marshfield Clinic Research Institute, Marshfield, WI, US; 3 Kaiser Permanente Washington Health Research Institute, Seattle, WA, US; 4 University of California Davis School of Medicine, Davis, CA, US; 5 Division of Research, Kaiser Permanente Northern California, Oakland, CA, US; 6 Department of Population Medicine, Harvard Medical School, Boston, MA, US; 7 Harvard Pilgrim Health Care Institute, Boston, MA, US; 8 Center for Health Research, Kaiser Permanente Northwest, Portland, OR, US; 9 Institute for Health Research, Kaiser Permanente Colorado, Aurora, CO, US; 10 Health Care Systems Research Network, Seattle, WA, US; 11 Retired

**Keywords:** Electronic Health Records, Delivery of Health Care, Quality Improvement, Health Services Research, Research Network

## Abstract

The Cancer Research Network (CRN) is a consortium of 12 research groups, each affiliated with a nonprofit integrated health care delivery system, that was first funded in 1998. The overall goal of the CRN is to support and facilitate collaborative cancer research within its component delivery systems. This paper describes the CRN’s 20-year experience and evolution. The network combined its members’ scientific capabilities and data resources to create an infrastructure that has ultimately supported over 275 projects. Insights about the strengths and limitations of electronic health data for research, approaches to optimizing multidisciplinary collaboration, and the role of a health services research infrastructure to complement traditional clinical trials and large observational datasets are described, along with recommendations for other research consortia.

## Introduction

**Figure 1:**
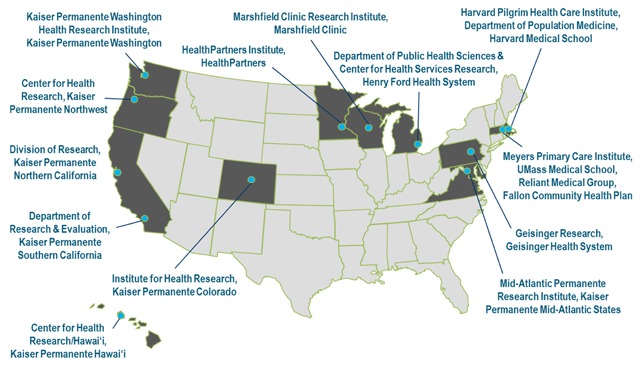
Cancer Research Network (CRN) Member Sites.

The Cancer Research Network (CRN) was founded in 1998 to increase the effectiveness of preventive, curative, and supportive interventions for major cancers, through a program of collaborative research based in community health care settings [1,2,3]. The CRN currently includes 12 research groups, each affiliated with a nonprofit integrated health care delivery system (Figure 1); collectively, these systems provide care to more than 12 million members. Member sites also belong to a larger organization of 18 health care delivery systems with in-house research centers known as the Health Care Systems Research Network (HCSRN; formerly Health Maintenance Organization Research Network, HMORN) [4,5]. HCSRN members have key features in common, including: a cadre of research scientists, comprehensive data resources mapped to a common data model (the Virtual Data Warehouse [VDW]), and a defined health care member population that generally reflects the demographics of their underlying communities. These attributes combine to provide a research resource to support multisite studies. This infrastructure review describes the rationale for, evolution of, and insights derived from the CRN over its 20-year history.

## Rationale for Establishing a Cancer Research Network in Integrated Health Care Delivery Systems

With one out of every three people in the United States developing cancer in their lifetime [6], we need a broad spectrum of approaches to counter the clinical, economic, and psychosocial effects of this pervasive illness. Historically, basic science has driven diagnostic and prognostic advances, and national clinical trials infrastructure (chiefly the National Cancer Institute [NCI] National Clinical Trials Network, previously the Cooperative Group Program) [7,8] has led to rapid progress toward optimal treatments. However, basic science and clinical trials do not fully address the array of questions about primary prevention, screening, treatment, long-term outcomes, and psychosocial sequelae of cancer, especially in community settings. Moreover, many adult cancer clinical trials have dismal accrual rates [7,9,10]. Thus, population studies that encompass epidemiologic and health services research, including observational studies and intervention studies outside an oncology clinical trials context, provide critical information about cancer’s broader impact and can assess the full range of determinants and consequences of this disease, as well as the experience of cancer patients who do not take part in clinical trials.

Before the CRN was established, the only major source of information about cancer-related health care utilization in the U.S. population was the Surveillance, Epidemiology, and End Results (SEER) cancer registry data linked with Medicare fee-for-service claims [11,12]. The SEER-Medicare database continues to be a widely-used resource; however, it has several limitations. Notably, SEER-Medicare provides information primarily for Medicare beneficiaries ages 65 years and older. Also, since health care utilization data are mostly based on Medicare claims, SEER-Medicare does not capture many details of care (e.g., results of tissue examinations and laboratory tests, specific chemotherapeutic agents administered and their doses, patient vital signs) or contextual information about the health system setting.

Cognizant of this, the NCI, in partnership with HMORN leaders, created the CRN to generate and provide standardized, interoperable sources of data covering cancer prevention, screening, diagnosis, treatment, survivorship, and end-of-life care across the entire age spectrum for defined populations. Doing research within a health care environment allowed not only for basic descriptive studies, but also for design and testing of interventions to improve how care is delivered. Conducting multisite research in a network also facilitated studying rare cancers by pooling data across multiple health care systems, and natural experiments to assess how differences in patient populations or health system factors influence care delivery.

The organizations that formed the original CRN have remained relatively stable in their structure, defined populations, and delivery system arrangements over the last two decades. Additionally, patients diagnosed with cancer in these health systems tend not to leave the health system to seek care elsewhere upon receiving a cancer diagnosis [13,14]. Along with population stability, the CRN has exhibited organizational stability, in that the sites developed and continue to maintain integral assets that comprise a robust infrastructure for cancer research. These assets include mature, comprehensive data resources, heterogeneous patient populations, administrative features that facilitate multisite collaboration such as reciprocity between Institutional Review Boards (IRBs) and subcontracting templates, and the opportunity to diffuse results from research into practice more effectively (since researchers are part of the parent health care system). Critically for cancer research, these organizations also have direct access to tumor registry data, either through their own internal registries, or through long-standing partnerships with SEER or state registries. This infrastructure enables projects that could not be readily undertaken in other settings.

## Evolution: The Changing Landscape of Cancer Care Delivery Research

Since the initial funding of the CRN, many advances have altered the landscape of cancer care delivery research. Basic science discoveries have yielded a better understanding of the processes by which genomic damage accumulates during the development of cancer. Subsequently, translational research has leveraged this knowledge to develop new cancer therapies, including drugs which target specific genetic mutations and therapies that activate a patient’s immune system to fight cancer [15,16]. Simultaneously, the development and widespread use of increasingly sophisticated tests for body imaging, cancer screening, and cancer staging have led to major changes in how and when cancers are diagnosed [17]. While in some instances these new screening, diagnostic, and therapeutic procedures have reduced cancer morbidity and mortality, in other cases technologies disseminate into routine care before there is evidence of efficacy (e.g., prostate specific antigen screening [18], robot-assisted surgery [19]). Furthermore, many new therapies are expensive, imposing significant financial burden even among insured patients, and exacerbating health disparities [20,21]. This trend is likely to continue as development of immunotherapy and biologics matures in the emergent precision medicine era.

These advances in cancer diagnosis and treatment are occurring in a rapidly changing health insurance and health care financing environment. In the last twenty years available health insurance products have become increasingly complex. The development of cost-sharing through high-deductible plans, for example, has increased the absolute costs paid by patients [22,23]. The passage of the Patient Protection and Affordable Care Act (ACA) of 2010 increased the number of insured individuals in the US by approximately 20 million [24], providing access to care for many who were previously uninsured, and mandated that certain preventive practices and screening tests be available without copayments [25]. The debate around potential modifications or repeal of the ACA, coupled with other proposed changes to federal health insurance programs, have led to a volatile landscape for both health care *and* health coverage, creating uncertainty and unpredictability for many patients undergoing cancer treatment. Consequently, especially for those undergoing cancer treatment, risks of financial toxicity and medical bankruptcy are increasing [26].

Evaluating the impact of the above changes is critical to continued progress in cancer control, particularly through research in community settings where most cancer patients are treated. Networks like the CRN are uniquely poised to examine these changes and evaluate the dissemination and comparative effectiveness of new screening/diagnostic tests and therapies and their impact on patient outcomes, including morbidity and mortality from the disease itself and impact on financial burden and other issues that affect quality of life.

Along with these changes in the health care landscape, concurrent advances in health information technology have facilitated research that taps into the huge volume of health data generated during routine clinical care. When the CRN was initially funded, research often included labor- and resource-intensive medical record abstraction to obtain information from handwritten chart notes. By 2006, however, all participating systems in the CRN had implemented electronic health record systems (EHRs) [27], which facilitated easier access to patient records for research purposes. Rapid growth of EHR systems has facilitated new types of clinical data collection, including patient-reported outcomes obtained via patient portals, and enabled the use of “big data” techniques such as natural language processing and other types of machine learning to extract data from unstructured clinical records in a more automated fashion, though validation of these methods can be complex and time-consuming [28,29]. More recently, digital health tools including smartphone and tablet apps, social media platforms, and wearable devices represent new frontiers in health care, especially consumer-centered care. These tools have the potential for novel data collection and better patient care, yet would benefit from robust evidence [30].

## Insights: Lessons Learned from 20 Years of the CRN

When the CRN was first created, few other consortia were conducting research in community-based health care settings, and most health services research was based on administrative claims data available from Medicare, Medicaid, or other health insurers. Over time, with increasing availability of health care data, many other research networks have been established. This section describes key insights from the CRN relevant to others who work in large, multicenter/team science settings. These insights apply to three areas: data, science, and culture.

### Data

One key innovation of the CRN was the development of the VDW, a distributed data model in which a set of common data standards (including variable names, definitions, formats, and data structures) is used across participating health systems to facilitate multisite research [1,31]. This allows each health system to maintain data locally and control data use, but enables a programmer at one site to write an analytic program that can be run at other sites, thereby minimizing or eliminating the need for duplicative effort and ensuring that analyses are consistent between sites. Each system’s VDW is populated with provider-, patient-, and encounter-level data extracted from EHRs and administrative and claims databases, as well as information from cancer registries. The VDW contains information about patient demographics, cancer and other diagnoses, health plan enrollment, vital signs, pharmacy dispensing, laboratory orders, utilization, and Census data [1,31]. With availability of data from EHRs, the VDW now also includes details of cancer infusion therapy data, not previously available in structured formats in community settings. The VDW provides the foundation for much of CRN’s research and has been the basis for data models used in other distributed networks such as the U.S. Food and Drug Administration’s Sentinel Initiative [32] and the Patient-Centered Outcomes Research Institute’s PCORnet [33,34,35,36].

Development of additional tools has enhanced VDW functionality and efficiency. The CRN has a query tool, CRNnet, to facilitate sharing programs and results for research preparation and quality assurance. CRNnet is the CRN’s installation of PopMedNet [37,38], an open-source platform to facilitate multisite research using distributed data. CRNnet’s secure distributed querying approach allows authorized users to issue standardized requests for summary-level data; these queries do not require development of SAS code, allowing for rapid responses to simple questions. Another innovation to enhance multisite research was the Cancer Counter [1], a data utility that allows approved users to obtain counts of primary tumors and numbers of cancer patients with specific tumor types using selected variables extracted from tumor registry files maintained by CRN health systems.

Despite these advances, there are important limitations of the distributed data approach. There was a persistent hope (and even misperception) that the CRN would become a turn-key, queryable data resource leveraging a defined population that any approved user could access. The appeal of a point-and-click tool to answer a myriad of health care delivery-related questions was compelling, yet the complexity of curating the data and collating and interpreting results remained a formidable challenge. Underlying differences between health systems—even those on the same EHR platform—necessitate deep local expertise about the system’s data, including contextual, temporal, and structural differences. Consequently, initial data extractions must be viewed carefully by users to avoid conducting analyses that yield invalid results.

For example, research on lung cancer screening at Kaiser Permanente Colorado (KPCO) was affected by difficulties in determining the denominator of screen-eligible patients. While documentation of smoking history is considered a vital sign that should be updated at every KPCO outpatient visit, researchers noted inconsistent and incomplete recording of this variable for potential study participants. Almost 20 percent had potentially inaccurate smoking status noted in both the EHR and VDW. Smoking status (including pack-years and time since quitting for former smokers) had not been updated for several outpatient visits, sometimes spanning years. Researchers determined that during clinic visits, clinic staff had not verbally re-assessed smoking status. Instead, EHR variables were “auto-filled” based on values derived from a previous visit. In response to this finding, KPCO researchers worked with clinical and operational leaders to quantify this issue within and across primary care medical offices, then worked with leadership to implement reminders and training within clinics. In this way, researchers’ discovery of data errors led to an operational, clinically relevant improvement, while also preventing use of incorrect data for research.

Another example comes from a CRN project evaluating trends in medical imaging utilization over time at seven CRN health care systems [39,40]. Quality checking of initial data pulled from participating health systems’ VDW files using a common SAS program identified several data issues. Undercounting of imaging exams occurred due to variations in local coding practices such as use of local radiology codes that did not reflect the tabulated Current Procedural Terminology (CPT), Healthcare Common Procedure Coding System (HCPCS), or International Classification of Diseases, ninth and tenth revisions (ICD-9 and ICD-10) codes, and/or variations and errors in how sites populated VDW fields. Additionally, overcounting occurred when one examination had different codes with nonmatching dates. (For example, inpatient procedures had ICD codes associated with hospitalization date and CPT codes associated with exam date.) Sites needed to resubmit data multiple times before quality issues were resolved, which was time-consuming. Accounting for the above issues greatly reduced variation in imaging utilization rates between sites. For example, utilization of head CT among enrollees aged 65 years or older initially varied 2.8- to 4.7-fold between sites with the highest versus lowest utilization; after addressing these issues, variation was reduced to a 1.3- to 2.0-fold difference (D. Miglioretti, unpublished data).

A third example comes from a current study by two CRN sites funded by a National Institutes of Health research project grant (R01CA207375). The study is assessing the comparative effectiveness of different clinical work-up strategies for incidentally-detected lung nodules, and includes an assessment of death following nodule detection and workup. On initial analysis, investigators noticed a more than 2-fold difference in mortality rates between two site cohorts using the same distributed VDW code. On additional review of VDW and medical records data, this discrepancy was determined to be due to different definitions of disenrollment dates among deceased health plan members. At one site, death dates generally corresponded to disenrollment dates, whereas at the other site, administrative processing resulted in death dates varying from disenrollment dates by up to 30 days. Initial code written at the first site inadvertently excluded many deceased members from the study cohort at the other site because the program falsely concluded that their disenrollment was not due to death. Adjusting the code to use death date in place of the recorded disenrollment date resulted in equivalent management of subject eligibility at both sites, and corrected the spurious discrepancy between site-specific mortality rates.

**Figure 2:**
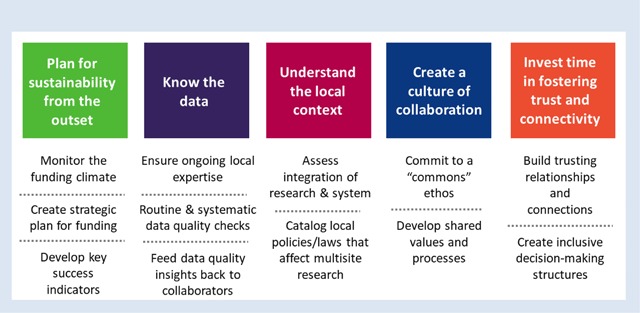
Key considerations for collaborative multisite research.

These examples highlight why point-and-click querying and automated epidemiology are insufficient for evidence generation and decision-making. Local variations in how data are collected, stored, and processed means that the validity of results from any project depends on repeated cycles of quality assurance and quality improvement, which ultimately requires local data and clinical expertise and validation from medical records. Even with significant investments in data curation, continued maintenance and quality checks are essential for any consortium [41]. Ideally, lessons learned through these quality checks are fed back to VDW site data managers to improve data quality and curation. A long-time CRN analyst once remarked, “Data get better with use,” which is particularly true in a federated data network. Though the VDW can be an efficient starting point for standardizing analytics (e.g., cohort extraction and aggregation), there is a need to confirm the accuracy of the analytic variables and approach (Figure 2).

### Science and Clinical Translation

Differentiating features of a health systems network linked through a collaborative data infrastructure include the ability to analyze a topic from patient, clinical, and system perspectives, and use the infrastructure to examine questions related to methodology, processes, and implementation. For instance, early CRN funding cycles were anchored by multisite studies with multilevel approaches. “HMOs Investigating Tobacco” (HIT), and “Detecting Early Tumors Enhances Cancer Therapy” (DETECT) studied, respectively, the organizational, clinician-level and patient-level factors that contribute to tobacco cessation and late-stage breast and cervical cancer occurrence. The HIT team sought to understand how nationally established tobacco cessation guidelines were implemented in nine CRN health systems, whether clinicians had adequate support for deploying the guidelines, and whether clinician-delivered tobacco cessation information affected patients’ satisfaction with care. The DETECT team sought to identify the extent to which late-stage breast and cervical cancers were attributable to breakdowns during the screening, detection, or treatment phases across six CRN sites. DETECT, in particular, was instrumental not only for clinically relevant findings [42,43,44] but also for promulgating a conceptual model [45] for studying and intervening on factors that influence cancer screening at multiple levels. As the CRN matured, its portfolio expanded to include affiliated grants and supplemental methodologic studies, and the CRN infrastructure was also leveraged to create new consortia [46,47,48,49].

Other studies leveraged the CRN’s large defined population, longitudinal data capabilities, and heterogeneity of the participating health systems to study the experience of cancer care. The CRN is unique in having sufficient data to study special populations, such as older women with breast cancer, a group for whom the most appropriate breast cancer care is still under-examined [50,51,52,53,54,55]. Similarly, a study of long-term psychosocial outcomes following prophylactic mastectomy benefited from the ability to identify women who received care from their health system for years after their procedure [56,57,58,59]. Diverse approaches to diagnosing and treating cancer patients has also enabled comparative effectiveness research evaluating deployment of advanced cancer therapies and associated outcomes [60,61], and utilization of KRAS and Lynch Syndrome testing to inform metastatic colorectal cancer treatment [62,63,64,65].

In a multicenter research environment, the potential exists for inefficiencies and duplicative processes. To alleviate this, CRN investigators documented inefficient processes and created a scientific infrastructure based on a cooperative ethos across the parent HCSRN. An integral example pertained to IRB review of study protocols. The aforementioned study of prophylactic mastectomy included a survey of women at six sites. Review of the same survey protocol across sites resulted in different decisions by the IRBs (e.g., determination of whether the study required full review, expedited review, or was exempt), and also introduced requirements for participant contact that varied by site [66]. Based partly on this experience, the CRN and HCSRN developed a reciprocal reliance model for multisite IRB review designed to mitigate variation and enhance efficiency [66,67,68].

A key takeaway from the CRN’s experiences addressing pragmatic questions in cancer control is that uptake of findings remains challenging. This is not unique to the CRN, as the research-to-practice gap is a well-worn topic [69,70,71]. However, since the CRN was in the unique position of being able to study these gaps and pursue potential interventions within health system settings, we hoped that our research would have widespread traction with health system leaders. This was not always the case, however. For example, the aforementioned DETECT study identified multiple opportunities for reducing late-stage diagnoses of breast and cervical cancer by enhancing patient follow-up and improving screening outreach, yet the health systems were not necessarily resourced or prepared to mobilize robust and rapid programs to address these opportunities. Similarly, the prophylactic mastectomy study identified informational needs for women considering this procedure, but the health systems’ competing priorities prevented them from deploying decision aids to address this unmet specialized need. Thus, despite proximity to health systems and applicable findings, research insights were unevenly translated at the system level. Thus, future consortia might consider aligning research questions with system leaders’ needs (Figure 2), which is consonant with the learning health system model [72,73].

To increase the chances of translating system-level interventions, planning for implementation and sustainability from the outset is imperative. For example, a study on cancer care quality from the perspective of oncologists, primary care clinicians, health policy researchers, and patients showed that patient navigation—already demonstrated to be effective in the cancer *screening* context—helped attenuate distress and support treatment decision-making during the diagnosis and treatment phases of a cancer patient’s journey [74]. This finding prompted a pragmatic trial on the role of nurse navigators for newly diagnosed breast and lung cancer patients. The positive results [75,76] led to permanent deployment of a nurse navigator in the health system after the study.

### Culture and Relationships

These CRN research successes depended on developing a culture of collaboration (Figure 2). While critical for any multidisciplinary, team-science endeavor, this culture has unique features for research conducted within health care systems. First, health care system researchers must incorporate studies into the clinical care context in which they are conducted. This requires establishing and maintaining relationships with clinical and operations leaders, both to design study protocols that can be incorporated into clinical workflows with minimal disruptions, and ideally, to ensure that research questions are selected to inform care delivery within the system. Matching research studies to clinical needs can be challenging. Although all health system stakeholders share the goal of improving care, research occurs at a slower pace than most quality improvement efforts by clinical and operations staff. Obtaining research funding is a slow process, hindering ability to address key clinical questions rapidly. Nevertheless, open dialogue between clinical, operations, and research staff can create an environment in which research informs quality improvement efforts and vice versa.

The CRN has used several approaches to enhance relationships between clinical, operations, and research staff. To address the issue of differing time horizons, in 2015, the CRN initiated a set of Rapid Analysis Projects (RAPs) that were intended to be conducted over a 6-month timeframe. To select these projects, a survey was distributed to practicing clinicians within the CRN health systems, asking about evidence gaps that impeded their daily practice. Survey respondents suggested research questions and a prioritization process classified the questions according to both importance and feasibility. A panel of research and clinical stakeholders selected projects based on questions viewed to be both important and addressable using VDW or other readily available data. While IRB approval and other challenges prevented RAPs from being conducted as quickly as desired, processes developed in response to delays are now embedded in CRN practice and should enable future RAP cycles to be conducted at a faster pace.

Another approach to creating an environment that stimulates relationships between clinical, operations, and research staff was the formation of the Translational Research in Oncology (TRIO) group by CRN Kaiser Permanente sites. TRIO’s goals include: providing a forum for Kaiser Permanente groups interested in improving cancer care and outcomes, minimizing duplication of analytic activities and bringing varied perspectives to addressing questions of interest, and identifying questions and projects of mutual interest to clinical, operational, and research staff. The group meets through quarterly webinars. Clinicians identify questions that impact patient care and may be amenable to EHR analysis, operations leaders prioritize questions to align with organizational goals and identify challenges and facilitators to improving care across the cancer spectrum, and researchers shape the questions and use them to design studies and analytic approaches.

Secondly, the CRN has cultivated collaborations with researchers at universities and cancer centers outside the CRN network. While CRN health systems have some clinician-researchers, they tend to be the exception rather than the rule, since clinical staff practicing within CRN health systems generally spend the majority of their time on patient care. External collaborations incorporate additional physician-scientists into CRN research programs, expanding the depth and breadth of expertise on research teams. Collaborations were also fostered through the CRN’s external advisory committee, which was comprised of leading cancer researchers based in academic institutions whose expertise included behavioral science, geriatric oncology, and clinical epidemiology. External investigators were key contributors to the medical imaging, breast cancer in older women, and nurse navigator projects described above. However, it is important to note that these partnerships can be labor-intensive; orienting outside investigators to the complexities of health systems and EHR data is time-consuming. Thus, strategically engaging investigators who complemented the CRN’s internal expertise proved to be the most effective approach to external collaboration.

Finally, building relationships with and training junior investigators has been critical for expanding the CRN’s research capacity. The CRN infrastructure provided new opportunities to conduct large population-based research studies within integrated health care systems, but required skills and levels of understanding that are not acquired in current scientific training programs. In response to this training gap, the CRN established the CRN Scholars Program [77,78] to assist junior investigators in: developing skills to collaborate with stakeholders in integrated health care systems, developing and using complex multisite data, and working in collaborative research teams anchored in delivery systems. The Scholars Program originated in 2007 as a 22-month training activity designed to help junior investigators at CRN sites develop research independence using CRN data and scientific resources. More recently, the Scholars Program extended to 26 months and broadened its reach to include junior investigators from internal *and* external institutions who are committed to conducting population-based cancer research.

During the program, each Scholar devoted 20 percent effort, receiving one-on-one and peer mentoring, participating in bimonthly webinars, and learning the analytic tools, methods, procedures, and processes for efficiently running multisite CRN studies. The training focus remains on preparing investigators to develop cutting-edge skills for conducting research in integrated health care delivery systems, fulfilling the goal of expanding the cadre of independent investigators committed to performing cancer research within the CRN.

Ultimately, successful collaborative relationships, whether among clinical, operations, and research staff, internal and external investigators, or senior and junior scientists, are enhanced by a commitment to shared scientific goals. A diverse network that encompasses a broad array of interests may experience times where investigators need to support others’ efforts on scientific questions that are not directly within their own research scope, applying a “commons” mindset. Adopting shared goals, even broad ones such as increasing adherence to screening or treatment guidelines, enhancing patient satisfaction with their care, or improving efficiency in care delivery, will help assure that priorities among researchers and between researchers and health systems are well-aligned. The CRN’s broad mission helped establish such an ethos.

## Conclusion

After continuous funding by the NCI since 1998, the CRN’s direct federal grant support will wind down in 2019, although numerous topic-specific collaborations will continue to flourish and perpetuate the CRN’s infrastructure and processes. Over 20 years of collaboration, the CRN has accrued important insights related to productive partnerships, opportunities and pitfalls in EHR data, and leveraging health system capabilities for research on improving cancer prevention, care, and control (Figure 2). Although we grouped our insights under the headings of data, science, and culture/relationships, these are clearly interdependent in a research network environment. Moreover, we believe many of these insights are pertinent to other multidisciplinary research collaborations, regardless of topical focus.

In the CRN’s history, given that studies encompassed prevention and early detection through treatment and end of life care, it was appealing to try to be “all things to all people”. We realized over time that this did not play to the CRN’s core strengths. For example, we had a diverse patient population cared for in community settings. Furthermore, CRN sites are substantial contributors to participation in oncology clinical trials, including the NCI’s Community Oncology Research Program [79]. However, we discerned through trial and error that our researchers’ priorities did not always align neatly with health system priorities, which meant that some research questions, despite their broad importance to the oncology community, were not suited to investigation within the CRN. In contrast, we were able to rapidly evaluate natural experiments, for example assessing diffusion of new therapies from controlled clinical trial settings to community practice [80,81]. We urge other research networks to be thoughtful about the attributes of the participating partners, and align the research agenda to the strengths and priorities of the participants.

While EHRs have facilitated “big data” research, they do not automatically produce research-ready data. The low marks that EHRs have earned for usability and interoperability [82,83] should also serve as cautions for researchers who use health record data. Our examples illuminate the importance of having project team members with intimate understanding of the underlying data sources who can apply knowledge about data idiosyncrasies in the research context, and compare differences across sites in a research consortium. A recent publication from the PCORnet data operations team [36] elaborates on the remarkable curation, validation and quality checking needed to ensure that data are research-ready. Laudably, the PCORnet [84], Sentinel [85], and HCSRN [86] data teams make their specifications and documentation public to help the research community, and have regular interactions to share lessons learned. However, since data sources are dynamic, it still behooves project teams to include subject matter experts who maintain current knowledge as data evolves.

At the time the CRN was first funded by the NCI in 1998, few others were conducting multi-site research based within health care delivery systems, and capacity-building funds were essential to develop the CRN’s research infrastructure. Over time, however, the advent of EHRs and development of new techniques to facilitate data extraction from clinical records for research purposes increased the depth and breadth of research that could be done, and further enabled numerous other health systems to develop their own research capabilities and shared infrastructures. Consequently, the CRN infrastructure grant evolved to emphasize support for collaboration, dissemination of knowledge, and investigator training over data development. In parallel, the need grew for the CRN’s component health systems, including their research arms, to explore other ways to maintain support for their research infrastructure. Sustainability of networks such as the CRN is dependent on organizational, sociopolitical, and financial exigencies, and ultimately requires the support and alignment of multiple stakeholders within each health care system. Furthermore, long-term network viability depends on continued evolution, such as promoting stronger connections to data science and informatics, inclusion of patients as part of the fabric of research teams, and attention to dissemination and implementation such that successful research has a higher potential for adoption. The CRN’s experience in developing data resources, creating a research program to improve patient care, and nurturing a culture of collaboration offers a practical roadmap for other research networks and health care organizations who want to use their collective resources and capabilities to improve health outcomes.
